# Maintenance inhaler therapy preferences of patients with asthma or chronic obstructive pulmonary disease: a discrete choice experiment

**DOI:** 10.1136/thoraxjnl-2019-213974

**Published:** 2020-07-06

**Authors:** Tommi Tervonen, Natalia Hawken, Nicola A Hanania, Fernando J Martinez, Sebastian Heidenreich, Ileen Gilbert

**Affiliations:** 1 Patient-Centered Research, Evidera, London, UK; 2 Department of Epidemiology, University Medical Center Groningen, University of Groningen, Groningen, The Netherlands; 3 Section of Pulmonary and Critical Care Medicine, Baylor College of Medicine, Houston, Texas, USA; 4 Division of Pulmonary Medicine, Weill-Cornell Medical Center, Cornell University, New York City, New York, USA; 5 AstraZeneca, Wilmington, Delaware, USA

**Keywords:** asthma, COPD exacerbations, inhaler devices

## Abstract

**Background:**

A variety of maintenance inhaler therapies are available to treat asthma and COPD. Patient-centric treatment choices require understanding patient preferences for the alternative therapies.

**Methods:**

A self-completed web-based discrete choice experiment was conducted to elicit patient preferences for inhaler device and medication attributes. Selection of attributes was informed by patient focus groups and literature review.

**Results:**

The discrete choice experiment was completed by 810 patients with asthma and 1147 patients with COPD. Patients with asthma most valued decreasing the onset of action from 30 to 5 min, followed by reducing yearly exacerbations from 3 to 1. Patients with COPD most and equally valued decreasing the onset of action from 30 to 5 min and reducing yearly exacerbations from 3 to 1. Both patients with asthma and patients with COPD were willing to accept an additional exacerbation in exchange for a 15 min decrease in onset of action and a longer onset of action in exchange for a lower risk of adverse effects from inhaled corticosteroids. Patients with asthma and COPD valued once-daily over twice-daily dosing, pressurised inhalers over dry powder inhalers and non-capsule priming over single-use capsules, although these attributes were not valued as highly as faster onset of action or reduced exacerbations.

**Conclusions:**

The most important maintenance inhaler attributes for patients with asthma and COPD were fast onset of symptom relief and a lower rate of exacerbations. Concerns about safety of inhaled corticosteroids and device convenience also affected patient preferences but were less important.

**Key messages**

**What is the key question?**

- What attributes of maintenance inhalers are most valued by patients with asthma or COPD?

**What is the bottom line?**

- The most important maintenance inhaler attributes for patients with asthma and COPD were fast onset of symptom relief and a lower rate of exacerbations.

**Why read on?**

- The findings highlight the importance of considering the patient perspective in selecting maintenance inhalers.

## Introduction

Inhaled corticosteroid (ICS)/long-acting beta-agonist (LABA) inhalers are routinely used for maintenance therapy in patients with asthma,^1^ and long-acting muscarinic antagonist (LAMA)/LABA and ICS/LABA inhalers are used for maintenance therapy in patients with COPD.^1 2^ A variety of inhalers are available, and although they have similar efficacy, their onset of action, adverse effects, dosing regimens, and other attributes differ.^1 2^

Patient-centric drug development and treatment decisions require understanding how patients value the different treatment attributes. Increasingly, decision-makers are promoting formal benefit-risk assessments for this purpose.^3^ Although qualitative research has provided some general insight into which treatment attributes are important,^4 5^ quantitative methods, especially discrete choice experiments (DCEs), can provide information about the patients’ willingness to make trade-offs among the attributes.^6^ In DCEs, respondents complete a series of questions in which they must choose between two treatments where there is a trade-off, for example, between efficacy and safety. In this way, the relative value of each treatment attribute can be discerned. DCEs have indicated that patients with asthma or COPD most value an inhaled therapy’s efficacy and safety, in addition to its ability to be used as a reliever medication, its convenience, an accurate dose counter, and low cost.^7–10^

Choosing an appropriate inhaler for patients has become more complex as the number of maintenance inhalers available has increased. This study used a DCE to establish which attributes of currently available ICS/LABA and LAMA/LABA combination inhalers are most valued by patients with symptomatic asthma or COPD, how willing they are to exchange one attribute for another, and how their choices are influenced by their disease status. It was a priori expected that patients with COPD place more weight on attributes related to treatment effectiveness and patients with asthma place more weight on attributes related to treatment convenience.

## Methods

### Overall study design

Following an initial literature review to identify relevant attributes of maintenance inhalers, focus groups were held separately for patients with asthma and COPD, to determine patient-generated positive and negative features of these treatments and to assess the relevance and appropriateness of the identified attributes to the burden of obstructive lung disease. Attributes previously reported in the literature but not spontaneously brought up by the patients were used to facilitate discussion during focus groups. A qualitative analysis of the information collected from the focus groups, along with consultation with clinical experts, was then used to select eight maintenance inhaler attributes to be included in a DCE. The DCE was pretested in a pilot study to refine the questionnaire and ensure that the selected attributes were meaningful to the patients.

### Participants

Patients with asthma or COPD included in a patient database of a recruitment agency (focus groups and pilot interviews) and in eight online access panels (main survey) were invited to participate by email or telephone. All participants had to be living in the USA and be able to speak, read, write, and understand English. Patients with asthma had to be aged ≥18 years with self-reported asthma and taking an ICS/LABA inhaler for at least 12 weeks. Patients with COPD had to be aged ≥40 years with self-reported COPD and taking a maintenance inhaler to treat COPD for at least 12 weeks. Patients with COPD were required to be symptomatic (COPD Assessment Test (CAT) score ≥10) or have had at least two exacerbations or one COPD-related hospitalisation in the past 12 months. Patients with COPD also had to be current or past smokers with a ≥10 pack-year history. Participants were excluded if they had a concurrent diagnosis of asthma and COPD. Patients with COPD were excluded from the pilot interviews and main survey if receiving triple therapy (ie, ICS/LABA+LAMA or ICS+LABA/LAMA) because fixed-dose combination triple therapies were not approved for use in the US at the time of this study.

Patients were compensated US$150 for participating in a focus group and US$100 for participating in an interview. Patients recruited from the access panels were compensated US$1.50–US$7.00 in accordance with a standing agreement with the online access panel.

### Focus groups

Separate focus groups for asthma (n=15) and COPD (n=22) were conducted in person in Dallas, Texas and Chicago, Illinois. Focus groups were conducted with the help of a semi-structured interview guide that focused on patients’ experiences with maintenance medications. An audio recording was made for transcription and qualitative analysis. Responses were used to determine which key issues patients were concerned with regarding the benefit, risks and other treatment attributes for maintenance therapy, and which potential attributes were appropriate and relevant. Narrative data were coded by two trained coders using a custom coding dictionary and analysed using ATLAS.ti V.7.5.9 (Scientific Software Development, Berlin, Germany). A third coder was consulted to resolve any disagreements.

### Qualitative pilot study

A qualitative pilot study was conducted online and by telephone using a semi-structured interview guide to ensure that patients could understand information in the DCE survey, including the attribute and level definitions, and that the burden of completing the survey was acceptable. An iterative process with two sets of five individual interviews each for asthma and COPD was used to refine the wording and organisation of the DCE questionnaire. Each interview lasted approximately 60 min and was audio recorded.

### Main study

At least 800 participants with asthma and 1250 with COPD were to be recruited to complete the DCE. Sample sizes were selected to obtain a sufficient representation of 150 patients using each of the different maintenance inhalers available, included as a recruitment quota. In addition, quotas were included to have ≥40% of each sex, ≥40% of patients with COPD within each of the <65 and ≥65 years age ranges, and ≥30% of patients with asthma in each of the 18–34, 35–64 and ≥65 years age ranges. Due to difficulty in meeting all recruitment quotas, the quotas were relaxed towards end of the recruitment period after an investigator review of the sufficient number of patients needed to complete the DCE for the relevant subgroups.

### Discrete choice experiments

Two DCEs, one for asthma and one for COPD, were administered as online surveys. Each consisted of 14 questions addressing maintenance inhaler attributes related to efficacy (onset of action and exacerbations per year), safety (5-year risk of osteoporosis) and non-clinical features (device type, dosing frequency, dose counters and priming). The COPD DCE also included an additional safety attribute, 5-year risk of pneumonia, because of concerns about a possible increased risk of pneumonia in patients with COPD treated with ICS.^11^

**Figure 1:**
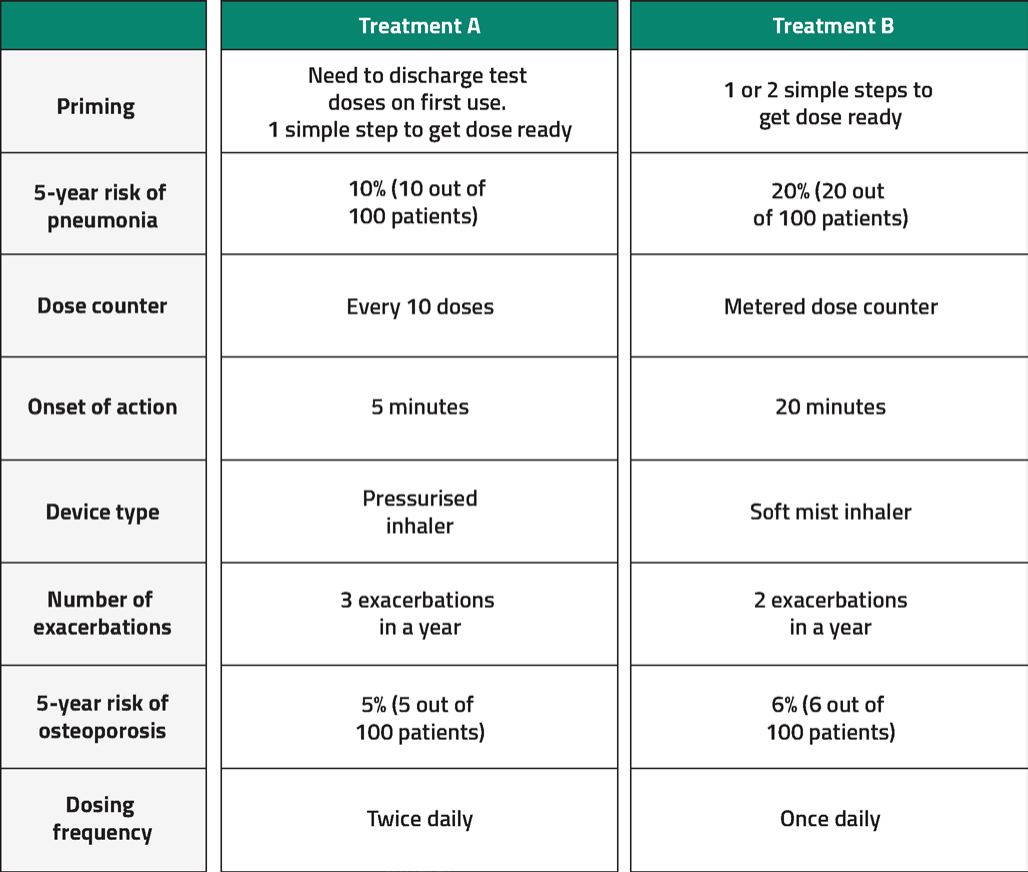
Example discrete choice experiment question.

Each attribute had two to four levels that were determined based on asthma and COPD treatment product characteristics, clinical expert feedback and a review of clinical data sources, published studies on patient perceptions and preference studies. An example question is shown in figure 1. To assess whether patients understood the discrete choice task presented in the questions and were responding appropriately to the choices, the DCE included a repeated question and a dominated-choice question (online supplementary figure 1), in which one of the medication choices was superior. For each participant, the order of attributes, choices and DCE questions were randomised, although for each participant, the attributes were presented in the same order within their survey.

10.1136/thoraxjnl-2019-213974.supp1 Supplementary data

Eligible participants were provided a brief description of the study’s purpose, instructions on how to complete the DCE questions and descriptions of the treatment attributes and levels. Patients with asthma completed the Asthma Control Questionnaire (ACQ),^12^ and patients with COPD completed the CAT.^13^ Before starting the online survey, participants completed a practice question. After completing the DCE, participants responded to a three-item subjective self-reported questionnaire to assess health literacy (Chew’s Set of Brief Screening Questions)^14^ and responded to a subset of items from the Numeracy Scale^15^ to ensure that they would be able to understand medical information and probabilities presented in the survey.

DCE data were analysed by estimating marginal utility values, which express preferences for changes in attributes using multinomial logit models (see online supplementary material). Given the ordinal nature of attributes, the interpretation of marginal utilities should be limited to significance and direction of the effect (ie, positive valuation vs negative valuation). To provide more detailed insight into patient preferences, three behavioural output measures were derived from the estimated marginal utilities: (i) relative attribute importance (RI) scores, which measure the share of variation in utility that can be explained by each attribute; (ii) maximum acceptable onset (MAO) of action, which measures trade-offs that patients are willing to make between improvements in other attributes and slower onset of action; and (iii) maximum acceptable exacerbations (MAE), which measures trade-offs that patients are willing to make between improvements in other attributes and average increase in yearly number of exacerbations.

Data were analysed separately according to patient diagnosis (asthma or COPD) and severity category (ACQ ≤0.75, >0.75 to <1.5, and ≥1.5 for asthma; CAT ≤20, >20 to ≤30, and >30 for COPD). Additional analyses were conducted to understand effects of age, sex and education level on patient preferences, while controlling for disease severity. Only fully completed surveys were included in the analysis. Stata V.15 (StataCorp, College Station, Texas, USA), R V.3.4 (R Foundation for Statistical Computing, Vienna, Austria) and Matlab V.R2017b (MathWorks, Natick, Massachusetts, USA) were used for the analyses. All statistical tests were two-sided and used a significance level of 0.05. SEs of RI, MAE and MAO were estimated using the delta method.

The internal validity of the DCE was assessed by measuring the time to complete the survey and the proportions of participants correctly answering the dominance test, correctly answering the repeated question, always choosing option A or B and always choosing the better alternative on one attribute (see online supplementary material). To avoid selection bias, no participants were excluded from the analysis based on their answers to internal validity assessments.^16 17^

## Results

### Participants

**Figure 2:**
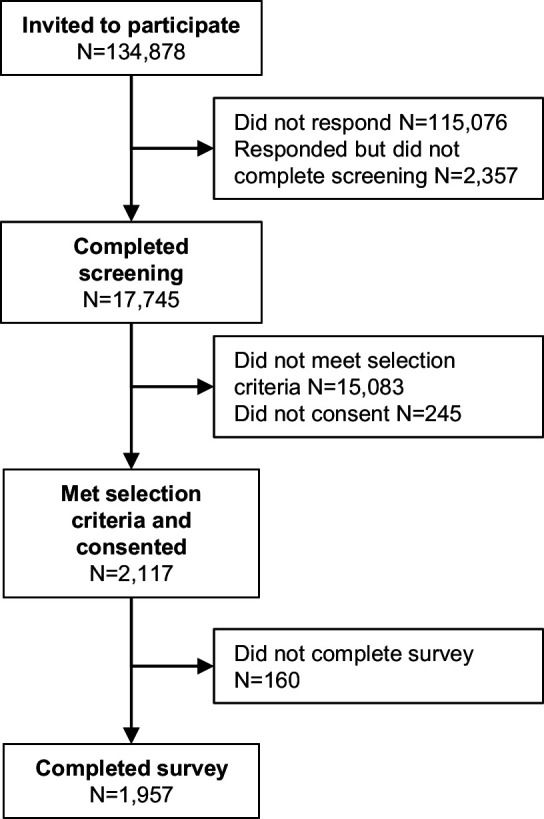
Disposition of participants in the main study.

The DCEs were completed by 810 patients with asthma and 1147 patients with COPD between May 30 and October 1, 2018 (figure 2). This includes the last seven patients who completed the qualitative pilot interviews because no significant changes were made to the survey afterwards. In both the asthma and COPD patient groups, most respondents were female (62% asthma, 57% COPD (p=0.06 for sex)) and white (76% for asthma, 87% for COPD), although patients with asthma were more often non-white than patients with COPD (p<0.0001) (table 1). The median age was 47 years for patients with asthma and 59 years for patients with COPD (p<0.0001 for mean age and age category). Compared with patients with COPD, patients with asthma more often were employed (34% vs 57%; p<0.0001 for overall employment status), had college education (72% vs 80%; p<0.0001 for overall educational status) and were married or living with a significant other (59% vs 62% asthma; p<0.0001 for overall current living/domestic situation). Most participants had high literacy (85% (n=1662)) and numeracy (88% (n=1729)) (online supplementary table 2).

**Table 1:** Demographic characteristics of participants

Characteristic	Asthma	COPD	P value*
	(n=810)	(n=1147)	
Sex, n (%)			0.0600
Male	310 (38)	488 (43)	
Female	500 (62)	659 (57)	
Age (years), median (IQR)	47 (35 to 61)	59 (52 to 67)	<0.0001
Age group (years), n (%)			<0.0001
18–34	201 (25)	0 (0)	
35–64	441 (54)	780 (68)	
≥65	168 (21)	367 (32)	
Racial background, n (%)			<0.0001
White	618 (76)	996 (87)	
Black	100 (12)	90 (8)	
Asian	21 (3)	15 (1)	
Hispanic	25 (3)	22 (2)	
Other	46 (6)	24 (2)	
Employment status, n (%)			<0.0001
Employed, full-time	384 (47)	330 (29)	
Employed, part-time	80 (10)	61 (5)	
Homemaker	73 (9)	73 (6)	
Student	13 (2)	1 (0)	
Unemployed	31 (4)	35 (3)	
Retired	169 (21)	415 (36)	
Disabled	58 (7)	222 (19)	
Other	2 (0)	10 (1)	
Education, n (%)			<0.0001
Elementary/primary school	12 (1)	6 (1)	
Secondary/high school	140 (17)	308 (27)	
Some college	244 (30)	380 (33)	
College degree	264 (33)	314 (27)	
Postgraduate degree	144 (18)	128 (11)	
Other	6 (1)	11 (1)	
Current living/domestic situation, n (%)			<0.0001
Married/living with significant other	504 (62)	680 (59)	
Divorced/separated	108 (13)	215 (19)	
Widow	29 (4)	129 (11)	
Single	166 (20)	119 (10)	
Other	3 (0)	4 (0)	

*P values were calculated by χ^2^ test for categorical variables and by analysis of variance for continuous variables.

Only 27% of patients with asthma were considered well-controlled (ACQ score ≤0.75^12^) (table 2). Most patients with COPD (99%) had a CAT score ≥10 (median, 26), indicating at least a medium impact of symptoms.^13^ All patients with asthma were taking an ICS/LABA maintenance inhaler. For patients with COPD, the most common maintenance inhaler type was ICS/LABA (52%), followed by LAMA or LABA (29%) and LAMA/LABA (21%).

**Table 2:** Disease severity and current medications

Characteristic	Asthma	COPD
	(n=810)	(n=1147)
ACQ score*, median (IQR)	1.3 (1.0 to 2.0)	–
ACQ score category†, n (%)		
≤0.75 (well-controlled)	219 (27)	–
0.75–1.5	195 (24)	–
≥1.5 (inadequately controlled)	396 (49)	–
CAT score‡, median (IQR)	–	26 (20 to 32)
CAT score category§, n (%)		
1–9 (low impact)	–	11 (1)
10–20 (medium impact)	–	323 (28)
21–30 (high impact)	–	476 (41)
31–40 (very high impact)	–	337 (29)
Current asthma medication, n (%)		
Budesonide/formoterol (Symbicort) (any dose)	263 (32)	–
80/4.5 HFA	136 (17)	–
160/4.5 HFA	127 (16)	–
Fluticasone/salmeterol (Advair HFA or Advair Diskus) (any dose)	429 (53)	–
115/21 HFA	113 (14)	–
230/21 HFA	80 (10)	–
250/50 Diskus	203 (25)	–
500/50 Diskus	33 (4)	–
Mometasone/formoterol (Dulera) (any dose)	48 (6)	–
100/25 HFA	26 (3)	–
200/25 HFA	22 (3)	–
Fluticasone/vilanterol (Breo) (any dose)	136 (17)	–
100/25 Ellipta	70 (9)	–
200/25 Ellipta	66 (8)	–
Current COPD medication, n (%)		
LAMA or LABA	–	337 (29)
Umeclidinium (Incruse Ellipta)	–	31 (3)
Tiotropium (Spiriva)	–	274 (24)
Olodaterol (Striverdi Respimat)	–	11 (1)
Aclidinium (Tudorza Pressair)	–	19 (2)
ICS/LABA	–	593 (52)
Budesonide/formoterol (Symbicort HFA)	–	263 (23)
Fluticasone/salmeterol (Advair Diskus)	–	295 (26)
Fluticasone/vilanterol (Breo Ellipta)	–	87 (8)
LAMA/LABA	–	236 (21)
Glycopyrrolate/formoterol (Bevespi Aerosphere)	–	34 (3)
Umeclidinium/vilanterol (Anoro Ellipta)	–	134 (12)
Tiotropium/olodaterol (Stiolto Respimat)	–	65 (6)
Glycopyrrolate/indacaterol (Utibron Neohaler)	–	17 (1)

*Average from seven questions, each scored from 0 (no impairment) to 6 (maximum impairment).

†ACQ categories were defined as described by Juniper *et al*.^12^

‡Sum of eight items scored from 0 (least severe impact on the patient’s life) to 5 (most severe impact), resulting in a total score of 0 to 40.

§CAT scores categories were defined as described by Jones *et al*.^13^

ACQ, Asthma Control Questionnaire; CAT, COPD Assessment Test; HFA, hydrofluoroalkane; ICS, inhaled corticosteroid; LABA, long-acting beta-agonist; LAMA, long-acting muscarinic antagonist.

### Patient preferences for treatment attributes

**Figure 3:**
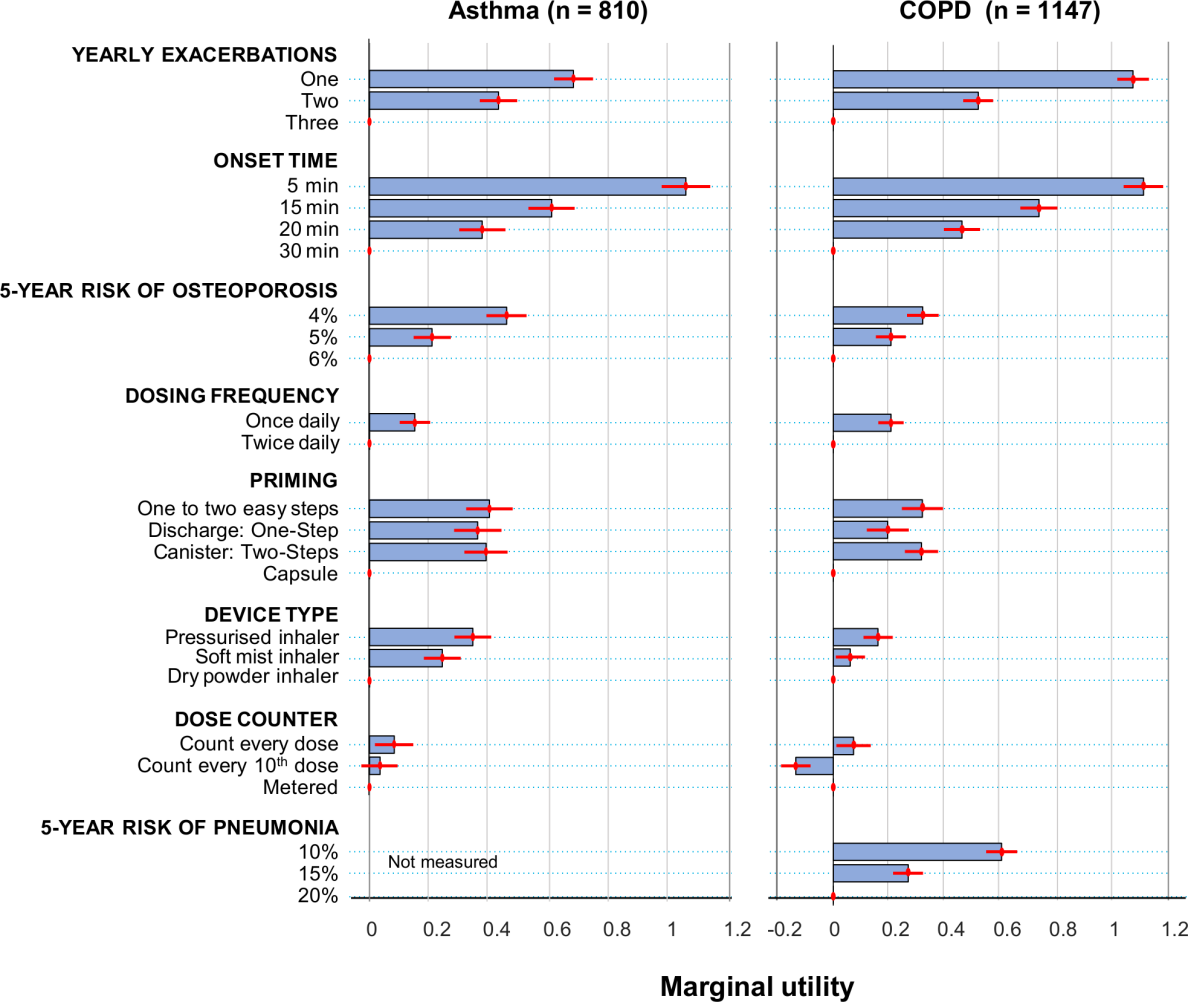
Overall preferences for inhaler attributes. Patient preferences were assessed by calculating marginal utilities, which measure how sensitive the respondents’ treatment preference is to changes between reference and non-reference levels of an attribute (see online supplementary table 3 for calculations). Values are means±95% CIs.

Patients with asthma most valued a decrease in medication onset of action from 30 to 5 min (RI=0.33), followed by a reduction in yearly exacerbations from 3 to 1 (RI=0.21), whereas patients with COPD most and equally valued a decrease in medication onset of action from 30 to 5 min (RI=0.28) and a reduction in yearly exacerbations from 3 to 1 (RI=0.27) (figure 3, online supplementary table 3). Overall, a faster onset of action and a reduction in yearly exacerbations were more highly valued than all other attributes. Both patients with asthma and patients with COPD also valued a decrease in the risk of treatment side effects (for asthma, marginal utility=0.21 (95% CI 0.15 to 0.27) for decreasing 5-year risk of osteoporosis from 6% to 5%; for COPD, marginal utility=0.21 (95% CI 0.15 to 0.26) for decreasing 5-year risk of osteoporosis from 6% to 5%; for COPD, marginal utility=0.27 (95% CI 0.21 to 0.32) for decreasing 5-year risk of pneumonia from 20% to 15%). In addition, patients with asthma and COPD valued pressurised inhalers over dry powder inhalers (marginal utility=0.34 (95% CI 0.28 to 0.41) for asthma and 0.16 (95% CI 0.11 to 0.21) for COPD), once-daily over twice-daily dosing (marginal utility=0.15 (95% CI 0.10 to 0.20) for asthma and 0.21 (95% CI 0.16 to 0.25) for COPD), and non-capsule priming methods over single-use capsules (marginal utility range, 0.36–0.40 for asthma and 0.20–0.32 for COPD). Most patients valued a dose counter that counts every dose over a metered dose counter (marginal utility=0.08 (95% CI 0.02 to 0.15) for asthma and 0.07 (95% CI 0.02 to 0.13) for COPD). Patients with COPD valued a metered dose counter over one that counts every 10 doses (marginal utility=−0.13 (95% CI −0.18 to −0.08)), whereas patients with asthma did not distinguish between the two dose counter types (marginal utility=0.03 (95% CI −0.03 to 0.10)).

### Willingness of patients to make trade-offs between attributes

Patient willingness to make trade-offs was assessed by estimating maximal acceptable time to onset of action and maximal acceptable yearly exacerbations (table 3, online supplementary tables 3 and 4), the two most valued inhaler attributes. Both patients with asthma and patients with COPD were willing to accept one extra exacerbation per year in exchange for a 15 min faster onset of action. For example, for a decrease in onset from 30 to 15 min, patients with asthma were willing to accept on average 1.8 more exacerbations per year and patients with COPD were willing to accept on average 1.4 more exacerbations per year. In exchange for decreasing the onset of action from 30 to 5 min, patients with asthma were willing to accept a greater increase in the yearly rate of exacerbations (on average 3.1 per year) than patients with COPD (on average 2.1 per year).

**Table 3:** Maximum acceptable exacerbations and maximal acceptable onset time by disease

Attribute	Level	Maximum acceptable exacerbations		Maximum acceptable onset time	
		Mean (95% CI)*		Mean (95% CI)†	
		Asthma (n=810)	COPD (n=1147)	Asthma (n=810)	COPD (n=1147)
Number of exacerbations	3 per year	–	–	Reference	Reference
	2 per year	–	–	10.1 (8.6 to 11.7) min	11.6 (10.3 to 12.9) min
	1 per year	–	–	16.0 (14.2 to 17.7) min	24.0 (22.3 to 25.7) min
Onset of action	30 min	Reference	Reference	–	–
	20 min	1.1 (0.85 to 1.3) per year	0.9 (0.7 to 1.0) per year	–	–
	15 min	1.8 (1.5 to 2.0) per year	1.4 (1.2 to 1.5) per year	–	–
	5 min	3.1 (2.7 to 3.4) per year	2.1 (1.9 to 2.2) per year	–	–
5-year risk of osteoporosis	6%	Reference	Reference	Reference	Reference
	5%	0.6 (0.4 to 0.8) per year	0.4 (0.3 to 0.5) per year	4.9 (3.5 to 6.4) min	4.5 (3.4 to 5.7) min
	4%	1.3 (1.1 to 1.5) per year	0.6 (0.5 to 0.7) per year	10.8 (9.2 to 12.4) min	7.1 (5.9 to 8.4) min
Dosing frequency	Twice daily	Reference	Reference	Reference	Reference
	Once daily	0.4 (0.3 to 0.6) per year	0.4 (0.3 to 0.5) per year	3.6 (2.4 to 4.8) min	4.6 (3.6 to 5.6) min
Priming	New capsule each time	Reference	Reference	Reference	Reference
	New canister+one step	1.1 (0.9 to 1.4) per year	0.6 (0.5 to 0.7) per year	9.2 (7.4 to 11.0) min	7.1 (5.7 to 8.4) min
	Discharge+one step	1.05 (0.8 to 1.3) per year	0.4 (0.2 to 0.5) per year	8.5 (6.6 to 10.4) min	4.4 (2.8 to 6.1) min
	One or two simple steps	1.2 (0.9 to 1.4) per year	0.6 (0.5 to 0.7) per year	9.4 (7.5 to 11.3) min	7.2 (5.6 to 8.9) min
Device type	Dry powder inhaler	Reference	Reference	Reference	Reference
	Soft mist inhaler	0.7 (0.5 to 0.9) per year	0.1 (0.0 to 0.2) per year	5.6 (4.1 to 7.1) min	1.4 (0.2 to 2.6) min
	Pressurised inhaler	1.0 (0.8 to 1.2) per year	0.3 (0.2 to 0.4) per year	8.1 (6.5 to 9.6) min	3.6 (2.4 to 4.8) min
Dose counter	Metered dose counter	Reference	Reference	Reference	Reference
	Every 10 doses	0.1 (−0.1 to 0.3) per year	−0.2 (−0.3 to −0.1) per year	0.7 (−0.7 to 2.2) min	−3.0 (−4.2 to −1.8) min
	Every dose	0.2 (0.1 to 0.4) per year	0.1 (0.0 to 0.2) per year	1.9 (0.4 to 3.4) min	1.6 (0.3 to 2.8) min
5-year risk of pneumonia	20%	–	Reference	–	Reference
	15%	–	0.5 (0.4 to 0.6) per year	–	6.0 (4.8 to 7.2) min
	10%	–	1.1 (1.0 to 1.2) per year	–	13.5 (12.2 to 14.9) min

*How many additional exacerbations a respondent was willing to accept for each of the attribute levels, relative to their respective reference level (see online supplementary table 4 for calculations).

†How many extra minutes of onset of action patients were willing to accept for each of the attribute levels, relative to their respective reference level (see online supplementary table 5 for calculations).

Patients with asthma were more willing than patients with COPD to accept a slower onset of action (10.8 min for asthma vs 7.1 min for COPD) in exchange for a reduced 5-year risk of osteoporosis from 6% to 4%, and they were willing to accept one extra exacerbation for a similar reduction in 5-year risk of osteoporosis. Patients with COPD were willing to accept a 13.5 min slower onset of action to reduce the 5-year risk of pneumonia from 20% to 10%. They were also willing to accept one extra exacerbation per year in exchange for this 10% reduction in the 5-year risk of pneumonia.

To obtain a pressurised inhaler instead of a dry powder inhaler, patients with asthma were more willing than patients with COPD to accept slower onset of action (8.1 min for asthma vs 3.6 min for COPD). In exchange for once-daily instead of twice-daily dosing, patients with asthma were willing to accept a 3.6 min slower onset of action, whereas patients with COPD were willing to accept a 4.6 min slower onset.

### Impact of disease status on patient preferences

**Figure 4:**
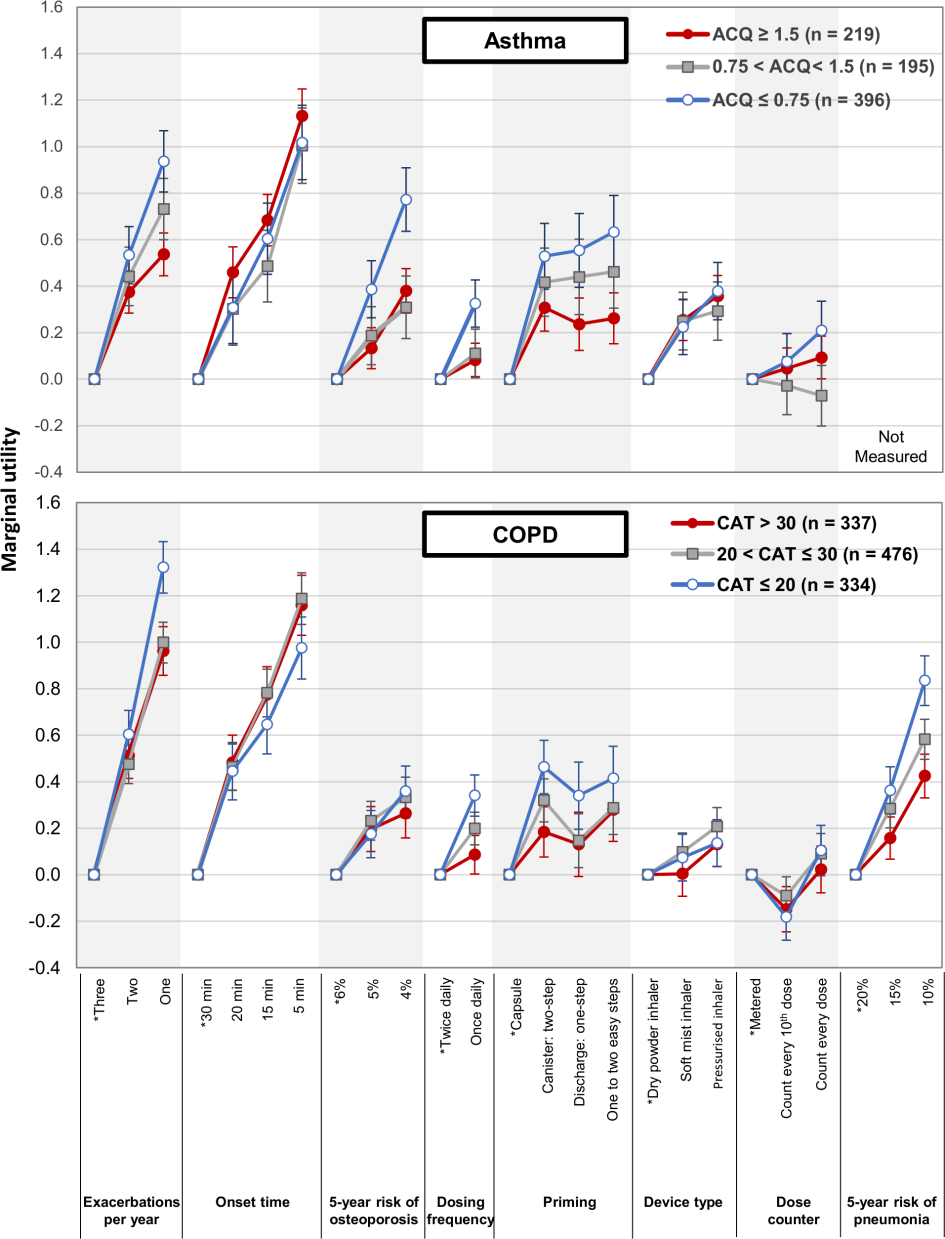
Impact of disease status on patient preferences. Patient preferences were assessed by calculating marginal utilities, which measure how sensitive the respondents’ treatment preference is to changes between reference and non-reference levels of an attribute (see online supplementary tables 6 and 7 for calculations). Values are means±95% CIs. Asterisk indicates reference level. ACQ, Asthma Control Questionnaire; CAT, COPD Assessment Test.

Patients with inadequately controlled asthma (ACQ score ≥1.5) considered faster onset of action (RI=0.39) to be more important than reduced exacerbations (RI=0.19) (figure 4, online supplementary tables 6 and 7). The difference in importance of faster onset of action and reduced exacerbations decreased as asthma control improved. Similarly, patients with COPD whose symptoms had the greatest impact on their health (CAT score >30) considered faster onset of action (RI=0.32) to be more important than a reduction in exacerbations (RI=0.27), whereas patients with the least symptom impact (CAT ≤20) considered a reduction in exacerbations (RI=0.27) more important than a faster onset of action (RI=0.20).

Reducing the risk of potential ICS adverse events was more important to patients with better disease status than to patients with worse disease status (for asthma, RI for 5-year risk of osteoporosis=0.18 for ACQ≤0.75, 0.10 for 0.75<ACQ<1.5 and 0.13 for ACQ≥1.5; for COPD, RI for 5-year risk of pneumonia=0.24 for CAT ≤20, 0.21 for 20<CAT <30 and 0.16 for CAT ≥30). Disease status affected preferences of patients with COPD for dosing frequency (marginal utility=0.34 (95% CI 0.25 to 0.43) for CAT ≤20, 0.20 (95% CI 0.13 to 0.27) for 20<CAT <30 and 0.09 (95% CI 0.00 to 0.17) for CAT ≥30).

Patient preferences were affected by age, sex and education (online supplementary tables 8 and 9). Key findings were that patients with college or postgraduate degrees valued exacerbation reduction and faster onset of action more than patients with less education; females placed more value on exacerbation reduction than males; and patients with asthma aged ≥65 years valued a dose counter that counts every dose more than those aged <65 years.

### Internal validity of the DCE

Of the 1957 participants, 88% (n=1731) answered the dominated-choice question correctly and 79% (n=1547) answered the repeated question correctly (online supplementary table 2). The dominated-choice question was incorrectly answered by 8% (n=93) of patients with COPD and 16% (n=133) of patients with asthma. Fewer than one in five participants (19% (n=364)) always chose the treatment for which one specific attribute had the most desirable level (eg, they always chose the treatment with the faster onset of action). The dominated-choice question was answered incorrectly by 33% of participants with low literacy and 41% with low numeracy but by only 8% of participants with high literacy and 8% with high numeracy.

## Discussion

DCE experiments conducted over the last decade have provided some information about which maintenance inhaler attributes are most valued by patients. Key inhaler attributes have included more symptom-free days,^9^ fewer adverse events,^9^ one-step dose preparation,^18^ ability to be used during breathing difficulties,^18^ dose counter accuracy,^18^ symptom control/not being disturbed during sleep,^10 18^ and low cost.^10^ However, the availability of new inhaled therapies and inhaler devices has made choosing an appropriate inhaler for patients more complicated. Furthermore, these studies have not examined the willingness of patients to trade-off between longer onset of action or additional exacerbations and improvements in other inhaler attributes.

This study, conducted in the USA in 2018, examined the relative importance of eight key maintenance inhaler attributes, including medication time to onset of action, number of exacerbations per year, risk of osteoporosis, risk of pneumonia (for COPD), dosing frequency, priming, device type, and dose counter. To capture the patient perspective, these attributes were selected from a previous patient focus group, coupled with a literature search and expert clinical advice.

Of the attributes examined, patients with symptomatic asthma or COPD most valued faster onset of action and reduced number of exacerbations. Both patients with asthma and patients with COPD were willing to accept increases in the rate of exacerbations in exchange for a faster onset of action, although patients with asthma appeared more willing than patients with COPD to make this exchange. Although patients valued these less than a faster onset of action and reduced exacerbations, both patients with asthma and patients with COPD valued a decrease in the risk of treatment side effects, pressurised over dry powder inhalers, once-daily over twice-daily dosing, more precise dose counters and non-capsule priming methods over single-use capsules. The study further found that disease status strongly influenced patient preferences: patients with more severe disease placed increased importance on a faster onset over a reduction in exacerbations.

Several assessments indicated internal validity of the data and adequate attention to the questions. In addition, efforts were made to include patients using all types of maintenance inhalers and with all relevant disease severities. Furthermore, the study was built to specifically capture patient preferences rather than healthcare providers’ expectations. Nonetheless, the study had some important limitations. Patient choices in hypothetical settings may not fully correspond to choices in real life, although recent research has shown that DCEs are able to predict choices.^19^ As with all surveys, DCEs are subject to framing and presentation effects.^20^ The study therefore followed conventional presentation formats and adhered to design guidelines, and the framing and presentation format of the DCE were tested in a qualitative pilot.^21^ Like other patient preference studies, we did not examine the relationship between patient preferences and treatment adherence.^22^ Another limitation is that clinical and sociodemographic characteristics were self-reported, although this is not expected to affect the conclusions. To limit the duration of the survey, we did not collect information on all possible variables, for example, we did not include questions on exacerbation history or LAMA add-on use in the asthma survey. Furthermore, the DCE study was limited to US patients registered in online access panels; the results may not be transferrable to patient populations in other countries or with different ethnical compositions. The asthma and COPD patient samples in this study were representative of US patient populations in terms of sex,^23^ and the COPD patient sample was representative in terms of ICS use,^24 25^ although 43% had a college degree, which is higher than the US average of 33.4% in 2016.^26^ Finally, to provide results relevant to prescribing clinicians, this study did not analyse the role of factors other than disease status in preference heterogeneity. Future analyses should address whether disease status is a key driver of differences and whether other factors such as pneumonia history, use of psychoanaleptics or experience with different inhalers independently affect preferences in these patient populations.

The current findings add substantially to the evidence on the preferences of patients with asthma and COPD for treatment attributes. Initial qualitative research indicated that safety and treatment costs are important to patients with asthma or COPD,^4 5^ and more recently, DCEs have indicated that patients with asthma and COPD highly value efficacy and safety and other attributes of inhaled treatments, such as use as rescue medication, convenience, rapid onset of action, accuracy of dose counter, and low cost.^8–10 18^ The current study was the largest DCE to date to explore preferences of patients with asthma and COPD for attributes of inhaled therapies, the first to consider adverse events due to long-term ICS use and one of the few to examine the influence of disease severity on preferences. The study also assessed device characteristics in greater detail than previous DCEs.

A potential limitation of this study is that it did not include nebulisers because most of the inhaler device attributes are difficult to measure for nebulisers. Thus, understanding why some patients prefer nebulisers over inhalers would require a different study design. Also, as per GINA^1^ and GOLD,^2^ nebulisers are not the preferred choice for the general obstructive lung disease population. Another potential limitation is that cost was not included as an attribute in the DCE. Although focus group interviews indicated that patients care about out-of-pocket costs, cost was not included as an attribute because the study focused on clinical and inhaler attributes of maintenance medications and because out-of-pocket costs in the USA vary according to each patient’s insurance scheme.

The current study suggested that patients with asthma or COPD would prefer treatments with a faster onset of action, greater exacerbation reduction and containing an ICS with a lower risk of osteoporosis and pneumonia. This information can be helpful to payers and policy makers who must decide which maintenance inhalers to recommend and reimburse. Many prescription plans in the USA already offer patients a choice, and practising providers have options for peer-to-peer conversations with insurers to ensure that medical provider and patient views are considered in coverage decisions. The results also highlight the importance of considering the patient perspective in selecting maintenance inhalers, a recommendation also made in treatment guidelines^1 2^ and in a recent review of maintenance inhalers for asthma and COPD.^22^
