# Supplementary material for: Maintenance inhaler therapy preferences of patients with asthma or chronic obstructive pulmonary disease: a discrete choice experiment
Source: Thorax. 2020 Jul 6;75(9):735–43. doi: 10.1136/thoraxjnl-2019-213974 (PMC7476258; doi:10.1136/thoraxjnl-2019-213974)
Supplement: Supplementary data [file thoraxjnl-2019-213974supp001.pdf]

Supplemental Material

ATTRIBUTE LEVELS AND DEFINITIONS

Discrete choice experiment (DCE) attributes were defined based on key efficacy and risk outcomes relevant for asthma and chronic obstructive pulmonary disease (COPD) maintenance inhaler treatments as well as features characterizing the convenience of using these maintenance inhalers and out-of-pocket costs for maintenance medicine. Because the attributes that are most relevant to patients differ for asthma versus COPD (e.g., increased risk of pneumonia is only relevant for COPD patients), two unique DCEs were administered to patients with asthma and patients with COPD. The DCE attributes were selected based on a targeted literature review, opinions generated from focus groups consisting of patients with asthma (n=15) or COPD (n=22),[1] and consultations with clinical experts on the project team.

The attributes for this study span three domains: (1) efficacy, characterized by onset of action of the inhaled therapy and exacerbations per year; (2) safety, characterized by 5-year risk of osteoporosis, and for COPD patients, 5-year risk of pneumonia; and (3) non-clinical features of the inhaler related to convenience, characterized by device type, dosing frequency, dose counters, and priming. The attribute levels were constructed based on: asthma and COPD treatment product characteristics; clinical expert feedback; and a review of clinical data sources, published studies on patients' perceptions of asthma and COPD endpoints, and preference studies in patients with asthma or COPD. Definitions of attributes and attribute levels are provided in **Supplemental Table 1**.

Tervonen T, *et al. Thorax* 2020; 75:735–743. doi: 10.1136/thoraxjnl-2019-213974

Supplemental Table 1. Attribute and Level Definitions

Attribute	Attribute Description	Level	Level Description
Number of exacerbations	**For Asthma:** An asthma exacerbation, or asthma attack, is when your airways become swollen and the muscles around your airways contract. This may cause you to experience difficulty breathing, coughing, or wheezing. You will need to visit your health care provider or emergency room to get extra medication, such as oral steroids, and may require an overnight stay in the hospital.<br>**For COPD:** A COPD exacerbation is when you experience a "flare-up", or sudden worsening of respiratory symptoms, and become sick. You will need to visit your health care provider or emergency room to get extra medication, such as oral steroids or antibiotics, and may require an overnight stay in the hospital.	1 exacerbation per year	You experience one exacerbation in a year.
		2 exacerbations per year	You experience two exacerbations in a year.
		3 exacerbations per year	You experience three exacerbations in a year.
Onset of action	After you use your maintenance inhaler, it may take a few minutes before it starts working. With some inhalers, the medication will start working within just 5 minutes, but with others it may take up to 30 minutes. The amount of time it takes for your medication to begin working is called onset of action.	5 minutes	Your medication will begin to work within 5 minutes after using your maintenance inhaler.
		15 minutes	Your medication will begin to work within 15 minutes after using your maintenance inhaler.
		20 minutes	Your medication will begin to work within 20 minutes after using your maintenance inhaler.
		30 minutes	Your medication will begin to work within 30 minutes after using your maintenance inhaler.
Osteoporosis	Some maintenance medications increase your chance of getting osteoporosis. Osteoporosis is a disease that affects your bones. Your body may then lose bone mass and your bones may become weak	4% (4 out of 100 patients)	You have a 4% chance of experiencing a hip fracture within 5 years of using your inhaler.
		5% (5 out of 100 patients)	You have a 5% chance of experiencing a hip fracture within 5 years of using your inhaler.

Tervonen T, *et al. Thorax* 2020; 75:735–743. doi: 10.1136/thoraxjnl-2019-213974

Attribute	Attribute Description	Level	Level Description
	and break easily. As a result, you may experience a hip fracture.	6% (6 out of 100 patients)	You have a 6% chance of experiencing a hip fracture within 5 years of using your inhaler.
Dosing Frequency	Dosing frequency refers to the number of times per day your doctor recommends you use your maintenance inhaler. Some maintenance medications should be used once per day while others should be used twice per day (once in the morning and once in the evening, approximately 12 hours apart).	Once daily	You need to take one or two puffs of your maintenance inhaler once per day.
		Twice daily	You need to take one or two puffs of your maintenance inhaler two times per day - once in the morning and again in the evening
Priming	Before you use your maintenance medication for the first time, it may require you to insert a cartridge containing all the doses. Some inhalers require you to follow a few simple steps to get it ready each time you use them. Others require you to remove a capsule from separate foil packaging and inserting it into the device.	One or two simple steps to get dose ready	Each time you use your inhaler, you will need to do one or two simple steps to get it ready. These steps only include opening the device and pulling either one or two levers. The steps are the same for your first dose and all other doses.
		Need to discharge test doses on first use. One simple step to get dose ready.	The device requires you to discharge several doses into the air before you use it for the first time. Between each discharge of a test dose, you must shake the device for five seconds. Each time you use your inhaler for all other doses, you will just need to open the device.
		Need to insert canister on first use. Two steps to get dose ready.	The device requires you to go through several steps to get it ready before you use it for the first time. First, you will need to remove the base of the inhaler and insert a separate canister of the medicine into the inhaler and push down hard until it clicks into place. Then, you must reattach the base into place. Each time you use the inhaler, you need to keep it upright, turn the base, and open the cap.

Tervonen T, *et al. Thorax* 2020; 75:735–743. doi: 10.1136/thoraxjnl-2019-213974

Attribute	Attribute Description	Level	Level Description
		Need to insert a new capsule into inhaler each time to get dose ready.	Each time you use this inhaler, you will need to remove a capsule containing the medicine from a separate package, insert it into the inhaler, and then close the inhaler to get it ready for use. The steps are the same for your first dose and all other doses.
Device type	Maintenance inhalers deliver your medicine in two different ways: 1) One type of inhaler is pressurized, like an aerosol can. The medication is forced out and you inhale moderately deeply and somewhat slowly. 2) The other type of inhaler contains the medicine in dry powder. For dry powder inhalers, you take a fast, very deep breath.	Pressurized inhaler	These inhalers are pressurized and force the medication out when you depress the canister or push the button. When you use these, you must inhale at slow speed, moderately deeply.
		Soft mist inhaler	These inhalers gently release the medication out in a continuous liquid mist when you press the dose-release button. When you use these, you must inhale slowly and deeply as you press the button, continuing until your lungs are full.
		Dry powder inhaler	These inhalers are not pressurized and hold the medicine in a dry powder form. When you use these inhalers, you must inhale very fast and deeply.
Dose counter	Inhalers often come with a dose counter built in that tells you how much medicine you have left. Some dose counters are very precise and display the exact number of doses that are left. Some dose counters are very precise but tell you how many doses you have left in counts of 10. Other dose counters are in the form of a meter, displaying an approximate amount of medicine left and look similar to a gas gauge in a car.	Every dose	The dose counter displays exactly the number of doses left.
		Every 10 doses	The dose counter displays the number of doses left but counts only every 10 doses.
		Metered dose counter	The dose counter is displayed as a needle on a meter, much like you would see a gas gauge in a car. It moves after every dose.

Tervonen T, *et al. Thorax* 2020; 75:735–743. doi: 10.1136/thoraxjnl-2019-213974

Attribute	Attribute Description	Level	Level Description
Pneumonia (COPD patients only)	Some maintenance medications increase your chance of getting pneumonia. Pneumonia is a type of lung infection that can cause coughing, fever, shortness of breath, fatigue, weakness, nausea, vomiting, and diarrhea.	10% (10 out of 100 patients)	You have a 10% chance of getting pneumonia in the coming 5 years.
		15% (15 out of 100 patients)	You have a 15% chance of getting pneumonia in the coming 5 years.
		20% (20 out of 100 patients)	You have a 20% chance of getting pneumonia in the coming 5 years.

DOMINATED-CHOICE QUESTION

For the dominated-choice question, one choice option was at least as good or better than the other on all attributes (**Supplemental Figure 1**). This dominated-choice question was used to assess whether patients understand the discrete choice task and were responding appropriately to the choices. The dominated-choice question was presented as the last question of the DCE exercise.

Although the patient population overall preferred once-daily over twice-daily dosing (**Figure 3**), some individual patients might have preferred twice-daily over once-daily dosing. For such patients, the dominated-choice question in **Supplemental Figure 1** is inappropriate.

Tervonen T, *et al. Thorax* 2020; 75:735–743. doi: 10.1136/thoraxjnl-2019-213974

Supplemental Figure 1. Dominated-Choice DCE Question

Your doctor asks you to decide between two maintenance inhaler medications, Inhaler A and Inhaler B. The outcomes you will experience after using either Inhaler A or B are described in the table below.

Take a moment to review the outcomes produced by each treatment, and then indicate which treatment you would choose at the bottom of the table.

**Outcomes**	**Inhaler A**	**Inhaler B**
Number of exacerbations	3 exacerbations per year	1 exacerbation per year
Onset of action	30 minutes	15 minutes
Osteoporosis	4% (4 out of 100 patients) risk of hip fracture over 5 years	4% (4 out of 100 patients) risk of hip fracture over 5 years
Pneumonia	20% (2 out of 100 patients) risk of pneumonia over 5 years	10% (1 out of 100 patients) risk of pneumonia over 5 years
Dosing frequency	Once daily	Twice daily
Device type	Pressurized inhaler	Pressurized inhaler
Dose counter	Metered dose counter	Metered dose counter

DCE DESIGN

Using NGene version 1.1 (ChoiceMetrics, Sydney, Australia), separate D-efficient experimental designs were generated for the asthma and COPD surveys, applying directional priors when applicable. The designs consisted of 96 questions split into eight survey versions (blocks) with 12 choice tasks per block and four blocks each for asthma and COPD. For each choice task, patients were asked to choose between two unlabeled treatments (A and B) described by the treatment attributes. The design did not include constraints for level combinations. Although some attribute combinations do not occur with currently available inhalers, such as capsule

priming with pressurized inhaler type, no issues with level combinations were detected in the pilot interviews.

STATISTICAL ANALYSIS OF DCE DATA

The analysis of the DCE data followed random utility maximization theory, by assuming that within every choice task (t), each respondent (n) always chose the treatment (j) that resulted in the highest utility.[2-4]

Baseline utility of patients with asthma was defined as

$$u_{jnt}^{\text{asthma}} = \alpha_{jnt}^{\text{asthma}} + \beta_1^{\text{asthma}}\text{one\_exacerbation}_{jnt} + \beta_2^{\text{asthma}}\text{two\_exacerbations}_{jnt} + \beta_3^{\text{asthma}}\text{20min \_onset}_{jnt} + \beta_4^{\text{asthma}}\text{15min\_onset}_{jnt} + \beta_5^{\text{asthma}}\text{5min \_onset}_{jnt} + \beta_6^{\text{asthma}}\text{5\% \_osteoporosis\_risk}_{jnt} + \beta_7^{\text{asthma}}\text{4\%\_osteoporosis\_risk}_{jnt} + \beta_8^{\text{asthma}}\text{once\_daily}_{jnt} + \beta_9^{\text{asthma}}\text{canister}_{jnt} + \beta_{10}^{\text{asthma}}\text{discharge}_{jnt} + \beta_{11}^{\text{asthma}}\text{1to2\_easy\_steps}_{jnt} + \beta_{12}^{\text{asthma}}\text{soft\_mist}_{jnt} + \beta_{13}^{\text{asthma}}\text{pressurized\_device}_{jnt} + \beta_{14}^{\text{asthma}}\text{1to2\_easy\_steps}_{jnt} + \beta_{15}^{\text{asthma}}\text{count\_evey\_10th\_dose}_{jnt} + \beta_{16}^{\text{asthma}}\text{count\_evey\_dose}_{jnt} + \varepsilon_{jnt}$$

Baseline utility of patients with COPD was defined as

$$u_{jnt}^{\text{COPD}} = \alpha_{jnt}^{\text{COPD}} + \beta_1^{\text{COPD}}\text{one\_exacerbation}_{jnt} + \beta_2^{\text{COPD}}\text{two\_exacerbations}_{jnt} + \beta_3^{\text{COPD}}\text{20min \_onset}_{jnt} + \beta_4^{\text{COPD}}\text{15min\_onset}_{jnt} + \beta_5^{\text{COPD}}\text{5min \_onset}_{jnt} + \beta_6^{\text{COPD}}\text{5\% \_osteoporosis\_risk}_{jnt} + \beta_7^{\text{COPD}}\text{4\%\_osteoporosis\_risk}_{jnt} + \beta_8^{\text{COPD}}\text{once\_daily}_{jnt} + \beta_9^{\text{COPD}}\text{canister}_{jnt} + \beta_{10}^{\text{COPD}}\text{discharge}_{jnt} + \beta_{11}^{\text{COPD}}\text{1to2\_easy\_steps}_{jnt} + \beta_{12}^{\text{COPD}}\text{soft\_mist}_{jnt} + \beta_{13}^{\text{COPD}}\text{pressurized\_device}_{jnt} + \beta_{14}^{\text{COPD}}\text{1to2\_easy\_steps}_{jnt} + \beta_{15}^{\text{COPD}}\text{count\_evey\_10th\_dose}_{jnt} + \beta_{16}^{\text{COPD}}\text{count\_evey\_dose}_{jnt} + \beta_{17}^{\text{COPD}}\text{10\%\_pneumonia\_risk}_{jnt} + \beta_{18}^{\text{COPD}}\text{15\%\_pneumonia\_risk}_{jnt}) + \varepsilon_{jnt}$$

Tervonen T, *et al. Thorax* 2020; 75:735–743. doi: 10.1136/thoraxjnl-2019-213974

where α_{left} was the constant of the left alternative to control for left-right bias, $\beta_1$to β_{18} are marginal utilities (i.e. estimated preference parameters) and ε_{jnt} was an extreme value distributed error that allowed the function to be estimated in a multinomial logit model.

All attributes were dummy-coded. Reference levels are indicated in Supplemental Table 1. Each of the estimated marginal utilities measured respondents' sensitivity to deviations from the reference level of the corresponding attribute. The sign (+ or -) of a marginal utility denoted whether patients valued this deviation positively or negatively. Three behavioral output measures were calculated to explore the trade-offs that patients are willing to make:

1. Relative attribute importance (RI) was estimated as the range of the marginal utilities within an attribute (i.e., the difference between the highest valued attribute and the lowest valued attribute), divided by the sum of the attribute ranges. RI scores sum up to one across each attribute and measure variation in overall utility that can be explained by changes in each attribute.

2. The maximum acceptable exacerbations (MAE) measured how many additional exacerbations a respondent was willing to accept for each of the attribute levels, relative to their respective reference level. This measure was obtained by estimating the baseline utility function with the number of exacerbations being coded as linear (i.e. one marginal utility is estimated instead of β_1 and β_2). Each marginal utility was then divided by the negative of the obtained marginal utility of the number of exacerbations.

3. The maximum acceptable onset time (MAO) measured how many extra minutes of onset of action patients were willing to accept for each of the attribute levels, relative to their

respective reference level. This measure was obtained by estimating the baseline utility function with the onset of action being coded as linear (i.e. one marginal utility is estimated instead of β_3 to β_5). Each marginal utility was then divided by the negative of the obtained marginal utility of the onset of action attribute.

The linearity assumptions made by MAE and MAO were tested by estimating the baseline utility function and fitting a linear function through the marginal utilities of the dummy coded attributes. Linearity was rejected for an R^2 <0.9. Standard errors of MEA and MAO were obtained using the delta method.

ASSESSMENT OF INTERNAL VALIDITY

Internal validity indicators included:

- Not choosing the better option in the dominated-choice question
- Choosing a different answer in the repeated question
- Always choosing the alternative better on one of the attributes with unambiguous attribute ordering (all attributes apart from method of treatment)
- Always choosing A or B; in addition, the discrete choice models included a constant to assess biases that are not binary (i.e., choosing sometimes A, sometimes B, but choosing A more often than they should, based on the level differences)
- Assessing time to complete the DCE survey

Internal validity was assessed for the full sample and stratified by educational level and health literacy and numeracy. Level of education was dichotomized into a high and low score, whereby bachelor's and/or postgraduate degrees were defined as a high educational level and all other educational levels are defined as a low educational level. Health literacy and numeracy were dichotomized as recommended by their developers.[5, 6] Results are presented in **Supplemental Table 2**.

Tervonen T, *et al. Thorax* 2020; 75:735–743. doi: 10.1136/thoraxjnl-2019-213974

Supplemental Table 2. Internal Validity Assessment Results

Validity assessment	n (%) Overall (N=1957)	Asthma (N=810)	COPD (N=1147)	Low Literacy (N=295)	High Literacy (N=1662)	Low Numeracy (N=228)	High Numeracy (N=1729)	Low Education (N=1107)	High Education (N=850)
Dominance test									
Failed	226 (12)	133 (16)	93 (8)	98 (33)	128 (8)	94 (41)	132 (8)	157 (14)	69 (8)
Passed	1,731 (88)	677 (84)	1,054 (92)	197 (67)	1,534 (92)	134 (59)	1,597 (92)	950 (86)	781 (92)
Repeated question									
Failed	410 (21)	155 (19)	255 (22)	74 (25)	336 (20)	55 (24)	355 (21)	251 (23)	159 (19)
Passed	1,547 (79)	655 (81)	892 (78)	221 (75)	1,326 (80)	173 (76)	1,374 (79)	856 (77)	691 (81)
Always choosing A or B									
A	40 (2)	35 (4)	5 (0)	29 (10)	11 (1)	34 (15)	6 (0)	36 (3)	4 (0)
B	1 (0)	1 (0)	0 (0)	0 (0)	1 (0)	0 (0)	1 (0)	0 (0)	1 (0)
Neither (some A, some B)	1,916 (98)	774 (96)	1,142 (100)	266 (90)	1,650 (99)	194 (85)	1,722 (100)	1,071 (97)	845 (99)
Always choosing the alternative better on one attribute									
None	1,593 (81)	673 (83)	920 (80)	239 (81)	1,354 (81)	200 (88)	1,393 (81)	929 (84)	664 (78)
Exacerbations	160 (8)	58 (7)	102 (9)	27 (9)	133 (8)	11 (5)	149 (9)	75 (7)	85 (10)
Onset of action	150 (8)	52 (6)	98 (9)	21 (7)	129 (8)	9 (4)	141 (8)	71 (6)	79 (9)
Osteoporosis	31 (2)	26 (3)	5 (0)	5 (2)	26 (2)	7 (3)	24 (1)	15 (1)	16 (2)
Pneumonia	23 (1)	1 (0)	22 (2)	3 (1)	20 (1)	1 (0)	22 (1)	17 (2)	6 (1)
Time to complete survey (min:s)									
3:00-4:59	1 (0)	1 (0)	0 (0)	1 (0)	0 (0)	1 (0)	0 (0)	1 (0)	0 (0)
5:00-6:59	33 (2)	25 (3)	8 (1)	19 (6)	14 (1)	14 (6)	19 (1)	22 (2)	11 (1)
7:00-9:59	83 (4)	61 (8)	22 (2)	26 (9)	57 (3)	22 (10)	61 (4)	44 (4)	39 (5)
9:00-14:59	515 (26)	265 (33)	250 (22)	89 (30)	426 (26)	77 (34)	438 (25)	265 (24)	250 (29)
15:00-19:59	427 (22)	166 (20)	261 (23)	47 (16)	380 (23)	40 (18)	387 (22)	252 (23)	175 (21)
≥20:00	898 (46)	292 (36)	606 (53)	113 (38)	785 (47)	74 (32)	824 (48)	523 (47)	375 (44)

Abbreviation: COPD, chronic obstructive pulmonary disease

Tervonen T, *et al. Thorax* 2020; 75:735–743. doi: 10.1136/thoraxjnl-2019-213974

RESULTS OF MULTINOMIAL LOGIT MODELING

Results of multinomial logit modeling by disease are shown in **Supplemental Tables 3–5**, by asthma severity groups in **Supplemental Table 6**, and by COPD severity groups in **Supplemental Table 7**.

Supplemental Table 3. Multinomial Logit Results (Dummy Coding) by Disease

	Asthma (N=810)			COPD (N=1147)		
**Attribute/level**	**Marginal utility (coefficient)**	**95% CI**	**RI**	**Marginal utility (coefficient)**	**95% CI**	**RI**
Left alternative	0.16***	0.12, 0.20	-	0.02	-0.02, 0.06	-
Exacerbations						
3 per year	Reference level			Reference level		
2 per year	0.43***	0.37, 0.49	0.21	0.52***	0.47, 0.57	0.27
1 per year	0.68***	0.61, 0.74		1.07***	1.02, 1.13	
Onset time						
30 min	Reference level			Reference level		
20 min	0.38***	0.30, 0.45	0.33	0.46***	0.40, 0.53	0.28
15 min	0.61***	0.53, 0.68		0.74***	0.67, 0.80	
5 min	1.05***	0.97, 1.13		1.11***	1.04, 1.18	
5-year risk of osteoporosis						
6%	Reference level			Reference level		
5%	0.21***	0.15, 0.27	0.14	0.21***	0.15, 0.26	0.08
4%	0.46***	0.39, 0.52		0.32***	0.26, 0.38	
Dosing frequency						
Twice daily	Reference level		0.05	Reference level		0.05
Once daily	0.15***	0.10, 0.20		0.21***	0.16, 0.25	
Priming						
Capsule	Reference level			Reference level		
Canister: two steps	0.39***	0.32, 0.46		0.32***	0.26, 0.38	
Discharge: one step	0.36***	0.28, 0.44	0.13	0.20***	0.12, 0.27	0.08
One to two easy steps	0.40***	0.32, 0.48		0.32***	0.25, 0.39	
Device type						
Dry powder inhaler	Reference level			Reference level		
Soft mist inhaler	0.24***	0.18, 0.30	0.11	0.06*	0.01, 0.11	0.04
Pressurized inhaler	0.34***	0.28, 0.41		0.16***	0.11, 0.21	
Dose counter						
Metered	Reference level			Reference level		
Every 10th dose	0.03	-0.03, 0.10	0.03	-0.13***	-0.19, -0.08	0.05
Every dose	0.08*	0.02, 0.15		0.07*	0.02, 0.13	
5-year risk of pneumonia						
20%				Reference level		
15%	Not included			0.268***	0.21, 0.32	0.15
10%				0.603***	0.55, 0.66	
Log-likelihood	-6027			-8220		
McFadden's pseudo-R²	0.11			0.14		

Abbreviations: COPD, chronic obstructive pulmonary disease; RI, relative importance score; SE, standard error.
*P<0.05. **P<0.01. ***P<0.001.

Supplemental Table 4. Multinomial Logit Results (Continuous Exacerbation Coding) by Disease

	Asthma (N=810)				COPD (N=1147)			
**Attribute/level**	**Coefficient**	**95% CI**	**MAE**	**95% CI**	**Coefficient**	**95% CI**	**MAE**	**95% CI**
Left alternative	0.16***	0.11, 0.20	-	-	0.02	-0.02, 0.06	-	-
**Exacerbations (1 per year)**	-0.34***	-0.37, -0.31	-	-	-0.54***	-0.57, -0.51	-	-
Onset time								
30 min		Reference level				Reference level		
20 min	0.37***	0.29, 0.45	1.1	0.8, 1.3	0.46***	0.40, 0.53	0.9	0.7, 1.0
15 min	0.60***	0.53, 0.68	1.8	1.5, 2.0	0.74***	0.67, 0.80	1.4	1.2, 1.5
5 min	1.05***	0.97, 1.13	3.1	2.7, 3.4	1.11***	1.04, 1.18	2.1	1.9, 2.2
5-year risk of osteoporosis								
6%		Reference level				Reference level		
5%	0.21***	0.15, 0.27	0.6	0.4, 0.8	0.21***	0.15, 0.26	0.4	0.3, 0.5
4%	0.45***	0.38, 0.52	1.3	1.1, 1.5	0.32***	0.26, 0.38	0.6	0.5, 0.7
Dosing frequency								
Twice daily		Reference level				Reference level		
Once daily	0.15***	0.10, 0.20	0.4	0.3, 0.6	0.21***	0.16, 0.25	0.4	0.3, 0.5
Priming								
Capsule		Reference level				Reference level		
Canister: Two steps	0.38***	0.31, 0.46	1.1	0.9, 1.4	0.32***	0.26, 0.37	0.6	0.5, 0.7
Discharge: One step	0.36***	0.28, 0.44	1.1	0.8, 1.3	0.20***	0.12, 0.27	0.4	0.2, 0.5
One to two easy steps	0.40***	0.32, 0.47	1.2	0.9, 1.4	0.32***	0.25, 0.39	0.6	0.5, 0.7
Device type								
Dry powder inhaler		Reference level				Reference level		
Soft mist inhaler	0.24***	0.18, 0.31	0.7	0.5, 0.9	0.06*	0.01, 0.11	0.1	0.0, 0.2
Pressurized inhaler	0.34***	0.28, 0.41	1.0	0.8, 1.2	0.16***	0.11, 0.21	0.3	0.2, 0.4
**Dose counter**								
Metered		Reference level				Reference level		
Every 10th dose	0.04	-0.02, 0.10	0.1	-0.1, 0.3	-0.13***	-0.19, -0.08	-0.2	-0.3, -0.1
Every dose	0.08*	0.02, 0.15	0.2	0.1, 0.4	0.07**	0.02, 0.13	0.1	0.0, 0.2
5-year risk of pneumonia								
20%						Reference level		
15%		Not included			0.27***	0.21, 0.32	0.5	0.4, 0.6
10%					0.60***	0.58, 0.66	1.1	1.0, 1.2
Log-likelihood		-6033				-8220		
McFadden's pseudo-R²		0.11				0.14		

Abbreviations: COPD, chronic obstructive pulmonary disease; MAE, maximum acceptable exacerbations; SE, standard error.

BMJ Publishing Group Limited (BMJ) disclaims all liability and responsibility arising from any reliance placed on this supplemental material which has been supplied by the author(s)

*P<0.05. **P<0.01. ***P<0.001.

Supplemental Table 5. Multinomial Logit Results (Continuous Onset Coding) by Disease

	Asthma (N=810)				COPD (N=1147)			
**Attribute/level**	**Marginal utility (coefficient)**	**95% CI**	**MAO**	**95% CI**	**Marginal utility (coefficient)**	**95% CI**	**MAO**	**95% CI**
Left alternative	0.16***	0.12, 0.20	-	-	0.02	-0.02, 0.06	-	-
Exacerbations								
3 per year	Reference level				Reference level			
2 per year	0.43***	0.37, 0.49	10.1	8.6, 11.7	0.52***	0.47, 0.57	11.6	10.3, 12.9
1 per year	0.68***	0.61, 0.74	16.0	14.2, 17.7	1.07***	1.02, 1.13	24.0	22.3, 25.7
Onset time (min)	-0.04***	-0.05, -0.04	-	-	-0.04***	-0.05, -0.04	-	-
5-year risk of osteoporosis								
6%	Reference level				Reference level			
5%	0.21***	0.15, 0.27	4.9	3.5, 6.4	0.20***	0.15, 0.26	4.5	3.4, 5.7
4%	0.46***	0.39, 0.52	10.8	9.2, 12.4	0.32***	0.26, 0.38	7.1	5.9, 8.4
Dosing frequency								
Twice daily	Reference level				Reference level			
Once daily	0.15***	0.10, 0.20	3.6	2.4, 4.8	0.20***	0.16, 0.25	4.6	3.6,5.6
Priming								
Capsule	Reference level				Reference level			
Canister: two steps	0.39***	0.32, 0.46	9.2	7.4, 11.0	0.32***	0.26, 0.38	7.1	5.7, 8.4
Discharge: one step	0.36***	0.28, 0.44	8.5	6.6, 10.4	0.20***	0.12, 0.27	4.4	2.8, 6.1
One to two easy steps	0.40***	0.32, 0.48	9.4	7.5, 11.3	0.32***	0.25, 0.40	7.2	5.6, 8.9
Device type								
Dry powder inhaler	Reference level				Reference level			
Soft mist inhaler	0.24***	0.18, 0.30	5.6	4.1, 7.1	0.06*	0.01, 0.11	1.4	0.21, 2.6
Pressurized inhaler	0.34***	0.28, 0.40	8.1	6.5, 9.6	0.16***	0.11, 0.22	3.6	2.4, 4.8
Dose counter								
Metered	Reference level				Reference level			
Every 10th dose	0.03	-0.03, 0.09	0.7	-0.7, 2.2	-0.13***	-0.19, -0.08	-3.0	-4.2, -1.8
Every dose	0.08*	0.02, 0.14	1.9	0.4, 3.4	0.07*	0.01, 0.13	1.6	0.3, 2.8
5-year risk of pneumonia								
20%	Not included				Reference level			
15%					0.27***	0.21, 0.32	6.0	4.8, 7.2
10%					0.61***	0.55, 0.66	13.5	12.2, 14.9
Log-likelihood	-6737				-9540			
McFadden's pseudo-R²	0.11				0.14			

Abbreviations: COPD, chronic obstructive pulmonary disease; SE, standard error.

Tervonen T, *et al. Thorax* 2020; 75:735–743. doi: 10.1136/thoraxjnl-2019-213974

BMJ Publishing Group Limited (BMJ) disclaims all liability and responsibility arising from any reliance placed on this supplemental material which has been supplied by the author(s)

*P<0.05. **P<0.01. ***P<0.001.

Tervonen T, *et al. Thorax* 2020; 75:735–743. doi: 10.1136/thoraxjnl-2019-213974

Supplemental Table 6. Multinomial Logit Results (Dummy Coding): Asthma by ACQ Groups

	ACQ ≤0.75 (N=219)			0.75 <ACQ <1.5 (N=195)			ACQ ≥1.5 (N=396)		
**Attribute/level**	**Marginal utility (coefficient)**	**95% CI**	**RI**	**Marginal utility (coefficient)**	**95% CI**	**RI**	**Marginal utility (coefficient)**	**95% CI**	**RI**
Left alternative	0.02	-0.07, 0.10	-	0.10*	0.01, 0.19	-	0.28***	0.21, 0.34	-
Exacerbations									
3 per year	Reference level			Reference level			Reference level		
2 per year	0.53***	0.41, 0.66	0.22	0.44***	0.32, 0.57	0.25	0.38***	0.28, 0.47	0.19
1 per year	0.94***	0.80, 1.07		0.73***	0.60, 0.86		0.54***	0.45, 0.63	
Onset time									
30 min	Reference level			Reference level			Reference level		
20 min	0.31***	0.15, 0.46	0.24	0.30***	0.15, 0.46	0.34	0.46***	0.35, 0.57	0.39
15 min	0.60***	0.45, 0.76		0.49***	0.33, 0.64		0.68***	0.57, 0.79	
5 min	1.02***	0.86, 1.18		1.00***	0.84, 1.17		1.13***	1.02, 1.25	
5-year risk of osteoporosis									
6%	Reference level			Reference level			Reference level		
5%	0.39***	0.26, 0.51	0.18	0.19**	0.06, 0.31	0.10	0.13**	0.05, 0.22	0.13
4%	0.77***	0.64, 0.91		0.31***	0.17, 0.44		0.38***	0.28, 0.48	
Dosing frequency									
Twice daily	Reference level		0.08	Reference level		0.04	Reference level		0.03
Once daily	0.33***	0.22, 0.43		0.11*	0.01, 0.22		0.08*	0.01, 0.15	
Priming									
Capsule	Reference level			Reference level			Reference level		
Canister: two steps	0.53***	0.39, 0.67	0.15	0.42***	0.27, 0.56	0.15	0.31***	0.21, 0.41	0.11
Discharge: one step	0.55***	0.40, 0.71		0.44***	0.28, 0.60		0.24***	0.12, 0.35	
One to two easy steps	0.63***	0.48, 0.79		0.46***	0.31, 0.62		0.26***	0.15, 0.37	
Device type									
Dry powder inhaler	Reference level			Reference level			Reference level		
Soft mist inhaler	0.22***	0.11, 0.34	0.09	0.25***	0.12, 0.37	0.10	0.25***	0.17, 0.34	0.12
Pressurized inhaler	0.38***	0.26, 0.50		0.29***	0.17, 0.42		0.36***	0.27, 0.45	
Dose counter									
Metered	Reference level			Reference level			Reference level		
Every 10th dose	0.08	-0.05, 0.20	0.05	-0.03	-0.15, 0.10	0.02	0.05	-0.04, 0.13	0.03
Every dose	0.21**	0.08, 0.34		-0.07	-0.20, 0.06		0.09*	0.00, 0.19	
Log-likelihood	-1580			-1445			-2946		
McFadden's pseudo-R²	0.13			0.11			0.11		

Abbreviations: ACQ, Asthma Control Questionnaire®; RI, relative importance score; SE, standard error. *P<0.05. **P<0.01. ***P<0.001.

Supplemental Table 7. Multinomial Logit Results (Dummy Coding): COPD by CAT Groups

Attribute/level	CAT ≤20 (N=334)			20 <CAT <30 (N=476)			CAT ≥30 (N=337)		
	Marginal utility (coefficient)	95% CI	RI	Marginal utility (coefficient)	95% CI	RI	Marginal utility (coefficient)	95% CI	RI
Left alternative	-0.04	-0.11, 0.03	-	0.01	-0.05, 0.07	-	0.08*	0.02, 0.15	-
Exacerbations									
3 per year	Reference level			Reference level			Reference level		
2 per year	0.60***	0.50, 0.71	0.27	0.48***	0.39, 0.56	0.24	0.51***	0.41, 0.61	0.27
1 per year	1.32***	1.21, 1.43		1.00***	0.91, 1.09		0.96***	0.86, 1.07	
Onset time									
30 min	Reference level			Reference level			Reference level		
20 min	0.45***	0.32, 0.57	0.20	0.46***	0.36, 0.56	0.28	0.48***	0.36, 0.60	0.32
15 min	0.65***	0.52, 0.77		0.78***	0.68, 0.88		0.77***	0.65, 0.90	
5 min	0.98***	0.84, 1.11		1.19***	1.08, 1.30		1.16***	1.03, 1.29	
5-year risk of osteoporosis									
6%	Reference level			Reference level			Reference level		
5%	0.18***	0.07, 0.28	0.07	0.23***	0.15, 0.32	0.08	0.20***	0.10, 0.29	0.07
4%	0.36***	0.25, 0.47		0.33***	0.24, 0.42		0.26***	0.16, 0.37	
Dosing frequency									
Twice daily	Reference level		0.07	Reference level		0.05	Reference level		0.02
Once daily	0.34***	0.25, 0.43		0.20***	0.13, 0.27		0.09*	0.00, 0.17	
Priming									
Capsule	Reference level			Reference level			Reference level		
Canister: two steps	0.46***	0.35, 0.58	0.09	0.32***	0.28, 0.41	0.08	0.18***	0.08, 0.29	0.08
Discharge: one step	0.34***	0.20, 0.49		0.15*	0.03, 0.26		0.13	-0.01, 0.27	
One to two easy steps	0.41***	0.28, 0.55		0.29***	0.17, 0.40		0.28***	0.14, 0.42	
Device type									
Dry powder inhaler	Reference level			Reference level			Reference level		
Soft mist inhaler	0.07	-0.03, 0.17	0.03	0.10*	0.02, 0.18	0.05	0.00	-0.09, 0.10	0.04
Pressurized inhaler	0.14**	0.04, 0.24		0.21***	0.13, 0.29		0.13**	0.03, 0.23	
Dose counter									
Metered	Reference level			Reference level			Reference level		
Every 10th dose	-0.18***	-0.28, -0.08	0.04	-0.09*	-0.17, -0.01	0.02	-0.15**	-0.25, -0.05	0.04
Every dose	0.10	-0.00, 0.21		0.09*	0.00, 0.18		0.02	-0.08, 0.12	
5-year risk of pneumonia									
20%	Reference level		0.24	Reference level		0.21	Reference level		0.16
15%	0.36***	0.26, 0.46		0.28***	0.20, 0.37		0.16**	0.06, 0.26	

Tervonen T, *et al. Thorax* 2020; 75:735–743. doi: 10.1136/thoraxjnl-2019-213974

10%	0.83***	0.73, 0.94	0.58***	0.50, 0.67	0.42***	0.32, 0.53
Log-likelihood	-2299		-3423		-2445	
McFadden's pseudo-R²	0.17		0.14		0.13	

Abbreviations: CAT, COPD Assessment Test; COPD, chronic obstructive pulmonary disease; RI, relative importance score; SE, standard error.
*P<0.05. **P<0.01. ***P<0.001.

Tervonen T, *et al. Thorax* 2020; 75:735–743. doi: 10.1136/thoraxjnl-2019-213974

Supplemental Table 8. Multinomial Logit Results for Asthma by Age, Sex, Level of Education, and ACQ Score

	Coefficient (95% CI)					
**Attribute/level**	**Marginal utility**	**≥65 y vs. <65 y**	**Female vs. male**	**University degree or higher vs. less than university degree**	**0.75 < ACQ < 1.5 vs. ACQ ≤0.75 or ≥1.5**	**ACQ ≥1.5 vs. ACQ <1.5**
Left alternative	0.17 (0.13, 0.22)***					
Exacerbations						
3 per year	Reference level					
2 per year	0.36 (0.17, 0.54)***	0.13 (-0.03, 0.30)	0.14 (0.01, 0.27)*	0.13 (0.00, 0.26)	-0.08 (-0.26, 0.10)	-0.14 (-0.30, 0.20)
1 per year	0.66 (0.47, 0.85)***	0.14 (-0.03, 0.32)	0.21 (0.07, 0.35)**	0.21 (0.08, 0.35)**	-0.18 (-0.37, 0.01)	-0.35 (0.52, -0.18)***
Onset time						
30 min	Reference level					
20 min	0.19 (-0.04, 0.41)	-0.02 (-0.23, 0.18)	0.05 (-0.11, 0.21)	0.19 (0.03, 0.35)*	0.00 (-0.22, 0.23)	0.16 (-0.04, 0.36)
15 min	0.42 (0.19, 0.64)***	-0.08 (-0.28, 0.12)	0.13 (-0.03, 0.29)	0.25 (0.09, 0.41)**	-0.11 (-0.33, 0.11)	0.09 (-0.10, 0.29)
5 min	0.47 (0.23, 0.70)***	0.10 (-0.12, 0.31)	0.34 (0.17, 0.51)***	0.52 (0.35, 0.69)***	0.06 (-0.17, 0.30)	0.23 (0.02, 0.43)*
5-year risk of osteoporosis						
6%	Reference level					
5%	0.28 (0.09, 0.46)**	0.03 (-0.13, 0.20)	0.15 (0.02, 0.29)*	0.02 (-0.11, 0.15)	-0.19 (-0.37,-0.02)*	-0.25 (-0.42, -0.09)***
4%	0.66 (0.47, 0.86)***	0.01 (-0.17, 0.19)	0.09 (-0.05, 0.24)	0.11 (-0.03, 0.25)	-0.45 (-0.65, -0.26)***	-0.39 (-0.57, -0.22)*
Dosing frequency						
Twice daily	Reference level					
Once daily	0.19 (0.04, 0.34)*	0.10 (-0.04, 0.24)	0.05 (-0.06, 0.16)	0.14 (0.04, 0.25)**	-0.20 (-0.35, -0.05)	-0.20 (-0.34, -0.07)
Priming						
Capsule	Reference level					
Canister: Two-Steps	0.29 (0.08, 0.49)**	0.29 (0.10, 0.48)**	0.14 (-0.01, 0.30)	0.12 (-0.03, 0.27)	-0.07 (-0.28, 0.14)	-0.15 (-0.34, 0.03)
Discharge: One-Step	0.34 (0.10, 0.58)**	0.27 (0.06, 0.49)*	0.11 (-0.05, 0.28)	0.11 (-0.06, 0.28)	-0.07 (-0.30, 0.16)	-0.25 (-0.46, -0.05)*
One to two easy steps	0.47 (0.24, 0.71)***	0.08 (-0.13, 0.28)	0.06 (-0.10, 0.23)	0.19 (0.02, 0.35)*	-0.15 (-0.37, 0.08)	-0.34 (-0.54, -0.14)***
Device type						
Dry powder inhaler	Reference level					
Soft mist inhaler	0.11 (-0.07, 0.28)	0.04 (-0.12, 0.20)	0.13 (0.00, 0.26)*	0.06 (-0.07, 0.19)	0.03 (-0.14, 0.21)	0.03 (-0.12, 0.19)
Pressurized inhaler	0.25 (0.06, 0.43)**	0.08 (-0.08, 0.25)	0.17 (0.04, 0.30)*	0.06 (-0.07, 0.19)	-0.10 (-0.28, 0.08)	-0.03 (-0.19, 0.13)
Dose counter						
Metered	Reference level					
Every 10th dose	0.10 (-0.08, 0.28)	-0.02 (-0.19, 0.14)	-0.01 (-0.14, 0.12)	-0.02 (-0.15, 0.11)	-0.10 (-0.27, 0.08)	-0.04 (-0.20, 0.12)
Every dose	0.09 (-0.09, 0.28)	0.26 (0.10, 0.43)**	0.02 (-0.11, 0.16)	0.04 (-0.09, 0.17)	-0.27 (-0.46, -0.09)**	-0.07 (-0.23, 0.09)

Abbreviations: ACQ, Asthma Control Questionnaire®; CI, confidence interval

Tervonen T, *et al. Thorax* 2020; 75:735–743. doi: 10.1136/thoraxjnl-2019-213974

Supplemental Table 9. Multinomial Logit Results for COPD by Age, Sex, Level of Education, and CAT score

	Coefficient (95% CI)					
**Attribute/level**	**Marginal Utility**	**≥65 y vs. <65 y**	**Female vs. male**	**University degree or higher vs. less than university degree**	**20 < CAT < 30 vs. CAT ≤20 or ≥30**	**CAT ≥30 vs. CAT <30**
Left alternative	0.02 (-0.02, 0.06)					
Exacerbations						
3 per year				Reference level		
2 per year	0.43 (0.28, 0.59)***	0.02 (-0.11, 0.14)	0.18 (0.07, 0.30)**	0.16 (0.04, 0.27)**	-0.12 (-0.25, 0.02)	-0.09 (-0.25, 0.06)
1 per year	1.04 (0.88, 1.21)***	0.15 (0.02, 0.28)	0.23 (0.09, 0.33)***	0.23 (0.10, 0.35)***	-0.28 (-0.43, -0.13)***	-0.32 (-0,48, -0.16)***
Onset time						
30 min				Reference level		
20 min	0.40 (0.22, 0.58)***	-0.12 (-0.27, 0.03)	0.09 (0.04, 0.23)	0.16 (0.02, 0.30)*	0.00 (-0.16, 0.17)	-0.02 (-0.21, 0.16)
15 min	0.57 (0.38, 0.76)***	=0.17 (-0.32, -0.01)*	0.14 (0.00, 0.28)*	0.20 (0.05, 0.34)**	0.12 (-0.05, 0.29)	0.06 (-0.13, 0.25)
5 min	0.98 (0.78, 1.18)***	-0.27 (-0.43, -0.10)**	0.04 (-0.11, 0.19)	0.30 (0.15, 0.46)***	0.18 (0.0, 0.36)	0.06 (-0.14, 0.26)
5-year risk of osteoporosis						
6%				Reference level		
5%	0.18 (0.30, 0.33)*	-0.02 (-0.14, 0.11)	-0.03 (-0.14, 0.09)	0.03 (-0.08, 0.15)	0.07 (-0.07, 0.20)	0.02 (-0.13, 0.17)
4%	0.37 (0.20, 0.53)***	-0.08 (0.02, 0.24)	-0.10 (-0.22, 0.02)	0.03 (-0.09, 0.16)	0.00 (-0.14, 0.15)	-0.06 (-0.23, 0.10)
Dosing frequency						
Twice daily				Reference level		
Once daily	0.26 (0.13, 0.39)***	0.13 (0.02, 0.24)*	0.04 (-0.05, 0.14)	-0.02 (-0.11, 0.08)	-0.12 (-0.23, 0.00)	-0.20 (-0.33, -0.07)**
Priming						
Capsule				Reference level		
Canister: Two-Steps	0.42 (0.25, 0.59)***	0.11 (-0.03, 0.25)	-0.04 (-0.17, 0.09)	0.00 (-0.13, 0.13)	-0.12 (-0.27, 0.04)	-0.21 (-0.38, -0.05)*
Discharge: One-Step	0.41 (0.19. 0.63)***	-0.01 (-0.18, 0.17)	-0.03 (-0.19. 0.13)	-0.12 (-0.29. 0.04)	-0.20 (-0.40, -0.01)*	-0.20 (-0.42, 0.02)
One to two easy steps	0.52 (0.30, 0.73)***	-0.14 (-0.31, 0.03)	0.02 (-0.14, 0.17)	-0.08 (-0.24, 0.08)	-0.017 (-0.36, 0.02)	-0.19 (-0.40, 0.02)
Device type						
Dry powder inhaler				Reference level		
Soft mist inhaler	0.09 (-0.06, 0.24)	-0.01 (-0.13, 0.11)	0.04 (-0.07, 0.15)	-0.12 (-0.23, -0.01)*	0.02 (-0.11, 0.15)	-0.05 (-0.20, 0.10)
Pressurized inhaler	0.21 (0.06, 0.36)**	-0.01 (-0.13, 0.11)	-0.16 (-0.17, 0.05)	-0.11 (-0.22, 0.00)	0.07 (-0.07, 0.20)	0.00 (-0.15, 0.15)
Dose counter						
Metered				Reference level		
Count every 10th dose	-0.22 (-0.38, -0.07)**	0.01 (-0.11, 0.14)	0.01 (-0.10, 0.12)	0.04 (-0.07, 0.16)	0.11 (-0.02, 0.25)	0.05 (-0.10, 0.20)
Count every dose	0.02 (-0.14, 0.18)	0.09 (-0.04, 0.22)	0.03 (-0.08, 0.15)	0.05 (-0.07, 0.17)	0.01 (-0.13, 0.16)	-0.05 (-0.20, 0.11)
5-year risk of pneumonia						
20%				Reference level		
15%	0.42 (0.27, 0.57)***	-0.02 (-0.15, 0.10)	-0.08 (-0.19, 0.03)	0.00 (-0.12, 0.11)	-0.08 (-0.21, 0.06)	-0.21 (-0.36, -0.06)**
10%	0.86 (0.70, 1.02)***	0.05 (-0.08, 0.18)	-0.11 (-0.23, 0.01)	0.04 (-0.08, 0.16)	-0.023 (-0.37, -0.09)	-0.40 (-0.56, -0.24)***

Tervonen T, *et al. Thorax* 2020; 75:735–743. doi: 10.1136/thoraxjnl-2019-213974

BMJ Publishing Group Limited (BMJ) disclaims all liability and responsibility arising from any reliance placed on this supplemental material which has been supplied by the author(s)

Abbreviations: CAT, COPD Assessment Test; CI, confidence interval; COPD, chronic obstructive pulmonary disease

REFERENCES

1. Hanania NA, Hawken N, Gilbert I, et al. What Symptomatic Patients with Asthma and Chronic Obstructive Pulmonary Disease (COPD) Find Important in Their Maintenance Inhaler Therapy: A Focus Group Study. Poster C37. *Am J Respir Crit Care Med* 2018;197:A4863.
2. Manski CF, Lerman SR. The Estimation of Choice Probabilities from Choice Based Samples. *Econometrica* 1977;45(8):1977-88.
3. McFadden D. Conditional Logit Analysis of Qualitative Choice Behaviour. In: Zarembka P, ed. Frontiers in Econometrics. New York: Academic Press, New York 1974:105-42.
4. Thurstone LL. A law of comparative judgement. *Psycholog Rev* 1927;34:273-86.
5. Chew LD, Bradley KA, Boyko EJ. Brief questions to identify patients with inadequate health literacy. *Fam Med* 2004;36(8):588-94. [published Online First: 2004/09/03]
6. Lipkus IM, Samsa G, Rimer BK. General performance on a numeracy scale among highly educated samples. *Med Decis Making* 2001;21(1):37-44. doi: 10.1177/0272989X0102100105 [published Online First: 2001/02/24]
